# Rapid Estrogenic and Androgenic Neurosteroids Effects in the Induction of Long-Term Synaptic Changes: Implication for Early Memory Formation

**DOI:** 10.3389/fnins.2020.572511

**Published:** 2020-10-27

**Authors:** Alessandro Tozzi, Laura Bellingacci, Vito Enrico Pettorossi

**Affiliations:** Department of Experimental Medicine, University of Perugia, Perugia, Italy

**Keywords:** 17β-estradiol, testosterone, 5α-dihydrotestosterone, P450 aromatase, 5α-reductase, LTP, LTD, synaptic plasticity

## Abstract

Mounting experimental evidence demonstrate that sex neuroactive steroids (neurosteroids) are essential for memory formation. Neurosteroids have a profound impact on the function and structure of neural circuits and their local synthesis is necessary for the induction of both long-term potentiation (LTP) and long-term depression (LTD) of synaptic transmission and for neural spine formation in different areas of the central nervous system (CNS). Several studies demonstrated that in the hippocampus, 17β-estradiol (E2) is necessary for inducing LTP, while 5α-dihydrotestosterone (DHT) is necessary for inducing LTD. This contribution has been proven by administering sex neurosteroids in rodent models and by using blocking agents of their synthesis or of their specific receptors. The general opposite role of sex neurosteroids in synaptic plasticity appears to be dependent on their different local availability in response to low or high frequency of synaptic stimulation, allowing the induction of bidirectional synaptic plasticity. The relevant contribution of these neurosteroids to synaptic plasticity has also been described in other brain regions involved in memory processes such as motor learning, as in the case of the vestibular nuclei, the cerebellum, and the basal ganglia, or as the emotional circuit of the amygdala. The rapid effects of sex neurosteroids on neural synaptic plasticity need the maintenance of a tonic or phasic local steroid synthesis determined by neural activity but might also be influenced by circulating hormones, age, and gender. To disclose the exact mechanisms how sex neurosteroids participate in finely tuning long-term synaptic changes and spine remodeling, further investigation is required.

## Introduction

Sex neurosteroids are a group of cholesterol-derived molecules that are synthesized *de novo* within the central nervous system (CNS) where they are able to exert local effects (Baulieu, 1981; Baulieu and Robel, 1990). Among these, estrogens and androgens influence the general development of neural circuits, motor adaptation, learning, memory (Luine and Frankfurt, 2012), emotional behavior (Altemus, 2017), and cognition (Luine, 2014), and they are implicated in some psychiatric disorders (Bangasser and Valentino, 2014) modulating the function of different areas of the brain such as the hippocampus, the amygdala, the vestibular and cerebellar systems, and the nucleus striatum (Beauchet, 2006; Andreescu et al., 2007; Colciago et al., 2015; Bender et al., 2017; Diotel et al., 2018; Mohamad et al., 2018; Wagner et al., 2018; Dieni et al., 2020; Luine and Frankfurt, 2020).

These actions have a major role in the induction and maintenance of neural connections during development and the entire life-span by directing synaptogenesis, dendritic spine formation, and functional long-term potentiation (LTP) or long-term depression (LTD) of synaptic transmission.

Enzymes responsible for the biosynthesis of neurosteroids from cholesterol are present in the nervous system (Mensah-Nyagan et al., 1999; Giatti et al., 2019). Testosterone is synthesized through conversion of cholesterol into pregnenolone, a neurosteroid that is subsequently metabolized, leading to formation of 5α-dihydrotestosterone (DHT) or 17β-estradiol (E2) by the action of the 5α-reductase or P450 aromatase enzymes, respectively. The local concentration of these neurosteroids was found to be significantly higher in some areas of the CNS (e.g., hippocampus) in respect to blood, where they bind to specific estrogen receptors (ERα, ERβ, and multiple isoforms) or to androgen receptors (AR) (Mukai et al., 2006; Hatanaka et al., 2015). These receptors are largely distributed in the CNS and their activation can elicit diverse genomic and non-genomic cell functions.

Besides the genomic effects that occur within hours, leading to up- or downregulation of gene transcription, E2 and DHT, the most active metabolite of testosterone, can also activate rapid intracellular signaling pathways, acting within seconds or minutes via extranuclear membrane-associated forms of receptors (Kawato, 2004). In neurons, membrane effects of E2 may lead to the stimulation of different enzymes such as phospholipase C (PLC) and protein kinase C (PKC) that in turn stimulate the production of IP3 and the elevation of intracellular calcium levels. These molecules may rapidly change the function of neurons by acting as second messengers leading to the activation of further enzymatic cascades including the src kinase and the MAPK/ERK pathway (Evinger and Levin, 2005; Srivastava, 2012; Murakami et al., 2018). As in the case of estrogens, evidence has been accumulated to involve rapid responses to androgens, dependent or independent on ARs (Foradori et al., 2008). Many cellular responses to androgens are transcription independent; in fact, activated ARs are able to associate with molecular substrates in the cytoplasm and the inner layer of the cell membrane to activate intracellular kinase cascades (Soma et al., 2018).

The activation of neural network within the CNS might produce rapid behavioral changes by stimulating estrogenic or androgenic sex neurosteroid signaling and rapidly modulating the enzyme function involved in the induction of long-term synaptic plasticity. Accordingly, the consolidation of hippocampal memory might be facilitated within minutes after treatments with estrogenic and androgenic neurosteroids or with agonists of their receptors, enhancing the performance on hippocampal memory tasks, as reported in experiments performed with rats and mice (Luine et al., 2003; Aubele et al., 2008; Benice and Raber, 2009; Inagaki et al., 2010, 2012; Babanejad et al., 2012; Phan et al., 2012, 2015; Jacome et al., 2016).

Here, we will review evidence supporting the immediate involvement of the most neuroactive estrogenic and androgenic neurosteroids, namely E2 and testosterone/DHT, in the induction of neuronal synaptic plasticity, the capability of neuronal circuitries to modify their function in response to environment-triggered electric signals. The electrophysiological actions of E2 and DHT on long-term synaptic plasticity will be presented.

## Neural E2 Influences Hippocampal LTP

The role of E2 acting as a neurosteroid in memory formation has been described since the late 1980s and has been recently confirmed by different research groups (Ooishi et al., 2012; Di Mauro et al., 2015; Fester and Rune, 2015; Hasegawa et al., 2015; Lu et al., 2019; Tozzi et al., 2019). These studies were guided by the major general evidence that inhibition of E2 synthase (P450 aromatase) activity produced hippocampal-related memory deficits both in women and in female rodents (Bayer et al., 2015; Tuscher et al., 2016). They also pointed out that E2, synthesized in the CNS, regulates cognition and behavior independently of gender, assigning to the neural E2 signaling system a general modulator role of CNS function (Moradpour et al., 2006; Saldanha et al., 2011; Bailey et al., 2013, 2017; Tuscher et al., 2016; Fester et al., 2017). In the hippocampus, E2-mediated synaptic activity was found to be related with memory formation and early and late influence of E2 on long-term electrophysiological synaptic effects and on dendritic spine formation has been described in animal models (Kramar et al., 2009).

The question how E2 can regulate the physiology of neurons involved in memory is still a matter of debate. E2 can directly influence the electric membrane properties of neurons or it can affect synaptic transmission, with synaptic changes suggested to be strongly correlated to functional modifications of nervous system networks. For example, E2 administration changed very rapidly the neuronal excitability and the synaptic responses of hippocampal pyramidal neurons, also triggering both short- and long-term effects on glutamate-mediated signaling (Wong and Moss, 1991, 1992). These rapid effects, occurring a few minutes after administration of this neurosteroid, strongly suggest major non-genomic mechanisms of action for E2-dependent facilitation of LTP (Warren et al., 1995; Cordoba Montoya and Carrer, 1997; Good et al., 1999). Electrophysiological recordings of hippocampal rat slices evidenced that E2 facilitation of LTP is mediated by NMDAR currents with effects both in males and females (Foy et al., 1999), as confirmed by pharmacological inhibition of the E2- and NMDA-dependent LTP using GluN2B-containing NMDA receptor antagonists (Smith and McMahon, 2005, 2006). The action of the E2-mediated modulation of NMDAR-dependent LTP was found to involve several intracellular protein kinases and membrane-associated targets, as suggested by experiments where incubation of hippocampal slices with blockers of ERs, MAPK/ERK, PKA, PKC, PI3K, NR2B, or CaMKII did not allow the induction of LTP in the presence of known E2 concentrations (Hasegawa et al., 2015). However, most of the studies exploring the influence of hippocampal E2 on synaptic memory involved experimental protocols where E2 was applied exogenously, an approach that may have limited the possibility to determine the exact source of neural E2, leaving unsolved the question whether LTP induction in physiological conditions needs the presence of the E2 neurosteroid. An important advance in the knowledge on the E2 origin and the mechanism of E2-mediated LTP was provided by studies using an electrophysiological and pharmacological approach of investigation that involved the use of drugs inhibiting the E2 synthesis or blockers of the ERs in animal models. Electrophysiological experiments aimed at investigating local mechanisms of LTP induction by using blocking agents of E2 synthesis were first performed in brain slices of male rats containing the medial vestibular nuclei (Grassi et al., 2009). Here, it has been found that the acute pharmacological inhibition of P450 aromatase with letrozole or the acute blockade of ERs (Grassi et al., 2009) with ICI 182780 (Scarduzio et al., 2013) prevented the LTP induction. This approach was then extended to the hippocampus where the role of locally synthesized E2 was demonstrated in male rats by measuring the effect of blockade of the E2 synthesis or receptors on the synaptic field potentials evoked in the CA1 region (Grassi et al., 2011; Pettorossi et al., 2013a). In fact, the LTP amplitude was markedly reduced under block of E2 synthesis or receptors, suggesting a facilitatory role of E2 on LTP. Further experiments performed in single hippocampal pyramidal neurons evidenced that in most of the neurons of the CA1 region, E2 is necessary for inducing LTP because pharmacological ER blockade fully prevented this form of synaptic plasticity (Tozzi et al., 2019). These studies also allowed to explore the role of ER subtypes on LTP induction. The use of selective ERα and ERβ inhibitors demonstrated that these ERs are both involved, as the ERα blocker MPP and the ERβ blocker PHTPP were both individually able to reduce LTP and were able to fully prevent it when applied together (Tozzi et al., 2019). The role of E2 synthesis on hippocampal LTP was further explored in male rats with experiments inhibiting the P450 aromatase activity (Tanaka and Sokabe, 2012, 2013; Hojo et al., 2015) and more recently by proving that ERα stimulation is able to induce LTP (Clements and Harvey, 2020). The role of E2 in the LTP induction has also been confirmed in mice knock-out for the P450 aromatase of both sexes, providing direct evidence that in the hippocampus, neuron-derived E2 is able to rapidly regulate the P450 aromatase-dependent Akt-ERK and CREB-BDNF signaling, essential for normal expression of LTP and synaptic plasticity (Lu et al., 2019).

The influence of E2 on LTP induction was generally observed in the hippocampus of adult male rats; however, different research groups pointed to age-dependent effects, with E2-dependent LTP observed only in young animals (Foy et al., 1999; Bi et al., 2000; Hojo et al., 2008). The influence of E2 on LTP has been also hypothesized to be gender specific. When E2 synthesis was chronically inhibited with letrozole, the LTP induction was prevented in female rats but not in males. This gender-dependent effect was paralleled by reduction of spine formation (Vierk et al., 2012; Fester and Rune, 2015). However, in a subsequent paper, the same group provided evidence that P450 aromatase inhibition by letrozole, applied acutely, prevented LTP induction both in male and female animals, confirming a more general role of E2 in hippocampal LTP (Vierk et al., 2012).

## Modulation of P450 Aromatase Affects LTP Induction

The pharmacological prevention of hippocampal LTP by inhibition of the E2 synthesis or of the ERs activity suggests that E2 is required during the induction phase of LTP, when neuronal afferences are activated by electrical stimulation. P450 aromatase is expressed at hippocampal level and it has been shown that its activation depends on neuronal activity (Kimoto et al., 2001; Hojo et al., 2004, 2008; Balthazart et al., 2006). Whether the P450 aromatase activity is responsible for a tonic synthesis of local E2 or is dependent on neural phasic inputs has not been established yet. Experimental evidences show that different high- or low-frequency stimulations in the rat hippocampus may account for bidirectional synaptic plasticity, with the synthesis of estrogen and androgens directly implicated in synaptic potentiation or depression, respectively (Tozzi et al., 2019). For example, whereas the inhibition of P450 aromatase impedes LTP induced by high-frequency stimulation, the concomitant presence of exogenous E2 allows a full LTP expression in a dose-dependent manner (Di Mauro et al., 2015, 2017). Moreover, while low-frequency stimulation normally depresses synaptic transmission (LTD), in the presence of exogenous E2, it is able to induce LTP. These evidences confirm that in the rat hippocampus E2 is a key factor for eliciting LTP and suggest that different stimulating frequency patterns might modulate the P450 aromatase activity and the local E2 neo-synthesis to sustain LTP (Di Mauro et al., 2015, 2017).

**FIGURE 1:**
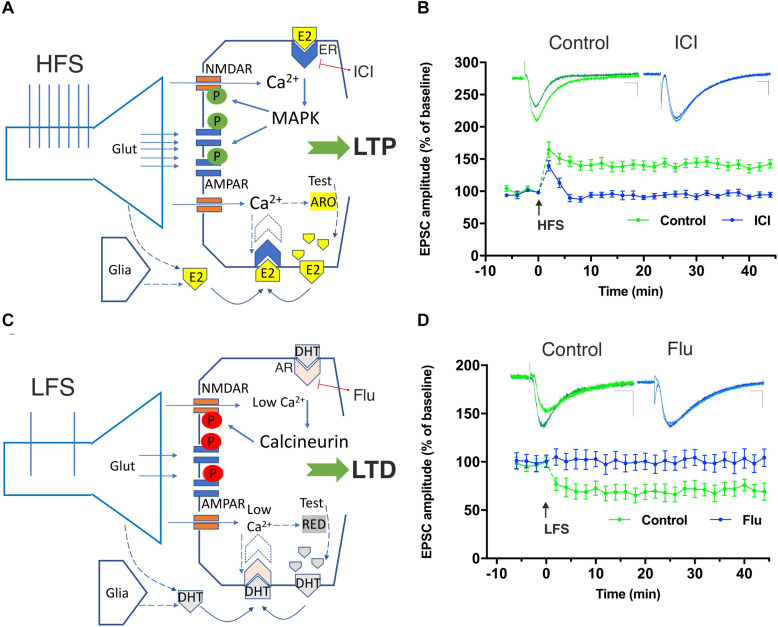
Involvement of neural 17β-estradiol (E2) and 5α-dihydrotestosterone (DHT), produced by testosterone (Test), in the induction of hippocampal high-frequency stimulation (HFS)–dependent long-term potentiation (LTP) and low-frequency stimulation (LFS)–dependent long-term depression (LTD). **(A)** The HFS-mediated glutamate (Glut) release stimulates large Ca^2+^ influx into dendritic spines through NMDA receptors. Ca^2+^ can activate calmodulin, PKC, and MAPK leading to phosphorylation (green symbols) of AMPA and NMDA glutamate receptors triggering LTP. Ca^2+^ is suggested to also stimulate P450 aromatase (ARO) activity increasing or decreasing the synthesis of E2, according to specific inputs to NMDARs and the Ca^2+^-dependent enzymes involved. After HFS, the synthesis of E2 may be locally enhanced and E2, released by presynaptic terminals and by the glia, may bind to membrane-gathered estrogen receptors (ERs) and to induce LTP through MAPK stimulation. **(B)** Graph showing the time course of the excitatory postsynaptic current (EPSC) amplitudes as percentage of the baseline, before and after an HFS is delivered to Schaffer collaterals in patch-clamp experiments from rat hippocampal CA1 pyramidal cells in control conditions (green time-course plot) and in the presence of the ER blocker ICI 182780 (10 μM, ICI) applied for the duration of the experiment (blue time-course plot). Note the LTP induced in control conditions but not in the presence of the ER blocking agent ICI. Superimposed representative traces showing an EPSC recorded before and 40 min after the HFS protocol in control conditions (green) and in the presence of ICI (blue). Scale bars: 10 ms; 50 pA. **(C)** LFS allows modest Ca^2+^ inflow into the postsynaptic neuron through NMDARs. Low Ca^2+^ levels are suggested to stimulate the 5α-reductase (RED)-dependent biosynthesis of DHT from testosterone (Lu et al., 2019). The locally synthesized DHT together with DHT released by presynaptic terminals and glia is suggested to activate membrane-gathered androgen receptors (AR) leading to the activation of calcineurin and to the dephosphorylation (red symbols) of different targets involved in LTD including the NMDAR. **(D)** Time-course graph of the EPSC amplitudes, before and after an LFS in control conditions (green time-course plot) and in the presence of the AR blocker flutamide (10 μM, Flu) applied for the duration of the experiment (blue time-course plot). Note the LTD induced in control conditions but not in the presence of the AR blocking agent Flu. Superimposed representative traces showing an EPSC recorded before and 40 min after the LFS protocol in control conditions (green) and in the presence of Flu (blue). Scale bars 10 ms; 50 pA. Modified by Tozzi et al. (2019).

Different research groups described a relation between changes of intracellular Ca^2+^ concentrations and P450 aromatase activity, implying the modulation of E2 synthesis (Balthazart et al., 2005; Fester et al., 2016). However, the precise mechanism by which neuronal electrical activity may lead to a Ca^2+^-dependent increase or decrease of E2 synthesis is still unknown. Exogenous glutamate application and K^+^-induced membrane depolarization have been suggested to trigger neuronal calcium-induced calcium release from intracellular Ca^2+^ stores, leading to enhancement of Ca^2+^ concentrations. This has been shown to lead to the activation of Ca^2+^-dependent kinases, P450 aromatase phosphorylation, and subsequent inhibition of E2 synthesis (Balthazart et al., 2001, 2003, 2005; Balthazart and Ball, 2006; Charlier et al., 2015; Fester et al., 2016). Moreover, a mechanism involving the action of extracellular E2 on NMDAR and on voltage-activated Ca^2+^ channels has been proposed to regulate E2 synthesis by influencing P450 aromatase activity (Zhao et al., 2005; Fester et al., 2016; Figure 1). Other investigators suggested a different scenario, describing a direct role of the NMDAR activation in the enhancement of intracellular Ca^2+^ with opposite effects on P450 aromatase activity. It has been reported that NMDAR activation triggers Ca^2+^-dependent kinases activity and increases E2 synthesis by P450 aromatase stimulation (Hojo et al., 2004; Figure 1). One possible explanation that might account for these opposite findings is the different experimental modality employed to stimulate neurons. Different glutamate receptors’ activation and velocity of Ca^2+^ entry into the cell may lead to opposite phosphorylation–dephosphorylation processes. The induction of LTP under the enhanced E2 availability was prevented by an inhibitor of calcium–calmodulin-dependent protein kinase II (CaMKII), suggesting that E2 is able to potentiate NMDA receptor function inducing an increase of postsynaptic Ca^2+^ concentration that in turn activates CaMKII leading to LTP induction (Hasegawa et al., 2015; Figure 1).

## Involvement of Neural E2 on Hippocampal LTD

The involvement of neural E2 on the scaling down of synaptic transmission, as in the case of LTD, has also been explored because the ability of E2 to modify synaptic plasticity via long-term depression may be an additional mechanism by which E2 can enhance learning and memory. In fact, E2 might act to improve memory by suppressing forgetting via an LTD-based mechanism.

Brain aging is generally associated to a decreased ability of memory processing and, in aged rats, to a facilitated hippocampal LTD. Accordingly, LTD amplitude was found to be larger in aged rats than in adults, and interestingly, treatments with E2 was reported to prevent this enhanced LTD in these aged animals but not in adults (Foy, 2001). Hippocampal LTD can be elicited by modest Ca^2+^ inflows entering into neurons via NMDA or metabotropic group I glutamate (mGluI) receptors (Shiroma et al., 2005). Interestingly, it has been found that the threshold for the induction of the NMDAR-dependent LTD could be enhanced or lowered by the stimulation of ERα or ERβ, respectively, confirming the role of ERs on LTD induction (Ooishi et al., 2012). Moreover, experiments also using ERα- and ERβ-knock-out mice confirmed that E2 may be able to rapidly enhance hippocampal LTD, presumably by a NMDAR- and ERα-dependent mechanism of action (Mukai et al., 2007; Murakami et al., 2015). However, electrophysiological experiments conducted in the presence of inhibitors of the E2 synthesizing enzyme or of the ERs suggested a minor role of E2 in hippocampal LTD because in the presence of these drugs, hippocampal LTD of male adult rats remained unchanged (Pettorossi et al., 2013a; Di Mauro et al., 2015, 2017).

## Androgens Affect Hippocampal LTP and LTD

It is well known that testosterone and its more active metabolite DHT attenuate mild cognitive impairment in men, suggesting a role of androgens in sustaining synaptic memory (Hogervorst et al., 2004; Pike et al., 2006; Kang et al., 2014; Pan et al., 2016). The concentration of these neurosteroids appears to be positively related with hippocampal synaptic spine density, suggesting that androgens are required for hippocampus-related cognitive performances (Moffat et al., 2002; Ooishi et al., 2012). The effects of androgens on the induction of hippocampal LTP and LTD has been recently examined by using drugs able to inhibit ARs or the 5α-reductase, the enzyme catalyzing the conversion of testosterone into DHT. Experimental findings demonstrated that in the rat CA1 hippocampal area, LTD is fully prevented under pharmacological blockade of either the AR or the 5α-reductase. These pharmacological actions, however, were unable to influence LTP in the same brain region and suggest that in the hippocampus, androgens sustain LTD but not LTP induction (Grassi et al., 2011; Grassi et al., 2013; Pettorossi et al., 2013a; Di Mauro et al., 2015; Di Mauro et al., 2017; Tozzi et al., 2019; Figure 1). Accordingly, ARs were found to be expressed in the hippocampus at the postsynaptic level (Tabori et al., 2005; Hatanaka et al., 2009), implying that ARs may participate in androgen-induced LTD.

Because testosterone may be converted either into E2 or DHT, depending on the prevalence, respectively, of the P450 aromatase or 5α-reductase synthesizing activity, it is suggested that the modulation of these biosynthetic pathways might in turn promote LTP or LTD in neurons (Di Mauro et al., 2015, 2017). Moreover, it is possible that the conversion of testosterone into DHT might limit the E2 neo-synthesis and the consequent possibility to induce LTP. Moreover, it has been hypothesized that a specific frequency of neuronal stimulation might drive hippocampal metabolism of testosterone toward conversion into E2 or DHT to sustain, respectively, LTP or LTD induction (Di Mauro et al., 2015).

Long-term depression in neurons may be based on different mechanisms, most of them implying an NMDAR-, mGluR- or endocannabinoid-dependent signaling. Ca^2+^ is suggested to play a role in LTD entering into neurons by glutamate receptors such as NMDARs during synaptic electric stimulations. Low-frequency stimulations (LFS) would produce low Ca^2+^ increases through NMDARs in the postsynaptic dendritic spines (Figure 1). After Ca^2+^-calmodulin formation, sequential activation of protein phosphatase 2B (calcineurin), dephosphorylation of inhibitor-1, activation of protein phosphatase 1 (PP1), and dephosphorylation of ser845 on the AMPAR subunit GluA1 would lead to internalization of AMPARs from the synapse, changes of the conductance properties of these receptors ultimately inducing LTD (Mulkey et al., 1994). However, how androgens are involved in LTD induction has been poorly investigated. It has been proposed that LFS-induced LTD is established in the hippocampus via DHT binding to synaptic ARs on delivery of LFS, leading to calcineurin activation and NMDAR suppression, resulting in a decreased presence or dephosphorylation of AMPARs (Hasegawa et al., 2015).

## Estrogenic and Androgenic Sex Neurosteroids Affect Vestibular LTP and LTD

The vestibular system is responsible for stabilizing the eyes and the body in space and is crucial for self-motion perception. It is involved in several plastic phenomena like the visuo-vestibular calibration (Lisberger and Miles, 1980), the vestibular compensation (Smith and Curthoys, 1989; Dutia, 2010), and the responsiveness to intense stimulation (Massot et al., 2012; Pettorossi et al., 2013b). Because LTP and LTD expression have been demonstrated in the vestibular nuclei and are likely involved in these adaptive responses (Grassi et al., 1995), the contribution of sex neurosteroids has been explored in medial vestibular nucleus (MVN).

Medial vestibular nucleus neurons express the E2 synthesizing enzyme P450 aromatase and both estrogen and androgen receptors. Specifically, immunoreactivity for ERα, ERβ, and AR have been found in these neurons, most of them co-localizing ERβ and AR (Grassi et al., 2013). Electrophysiological studies in rat MVN neurons showed that E2 affects synaptic transmission and neuronal excitability facilitating both the LTP of the primary vestibular afferents and the intrinsic membrane excitability (Grassi et al., 2007, 2009, 2010). Specifically, during LTP, E2 depresses the spontaneous action potential discharge in both regular (A type) and irregular (B type) discharging neurons, while the synaptic response to vestibular nerve stimulation is increased in B type neurons with a net effect of enhancement of the signal-to-noise ratio of synaptic response in these neurons, relative to resting activity of all MVN neurons. These combined effects may be necessary to specifically enhance the dynamic properties of neuronal activation of vestibular circuits. Interestingly, pharmacological inhibition of E2 synthesis by letrozole or antagonism of ERs by ICI 182780 was reported not only to prevent the induction of LTP but also to unmask LTD of synaptic plasticity, suggesting that the level of neural E2 is a key modulator of MVN neurons’ synaptic plasticity, being able to shift LTP into LTD according to the local availability of this neurosteroid (Grassi et al., 2009, 2010).

Because MVN neurons have been reported to also express androgens, it is plausible that androgens play an important role in LTD induction of synaptic plasticity also in MVN, as demonstrated in other brain regions such as the hippocampus. In fact, it has been shown that the pharmacological antagonism of ARs abolishes this form of synaptic plasticity in MVN neurons, as observed by electrophysiological recordings (Scarduzio et al., 2012, 2013). However, differently from what was observed in the hippocampus, the reduction of DHT synthesis from testosterone by pharmacological inhibition of 5α-reductase did not affect LTD, suggesting a more direct effect of testosterone and/or a greater sensitivity of MVN neurons to this neurosteroid with respect to DHT (Scarduzio et al., 2012, 2013).

## Neural E2 Affects Cerebellar LTP and LTD

The cerebellum participates with the vestibular system to most of the adaptation observed in the gaze stability with multiple synaptic plasticity mechanisms (Lisberger and Miles, 1980). In particular, together with the vestibular nuclei, it is responsible for the vestibulo-ocular reflex calibration (Hogervorst et al., 2004). In this encephalic structure, the encoding gain increase and decrease adaptation is thought to be mediated by LTP occurring at the parallel fiber–Purkinje cell synapses (PF-LTP) and by LTD (PF-LTD) at the same or different synapse subset, respectively (Hansel et al., 2001; Boyden and Raymond, 2003; Boyden et al., 2004; Coesmans et al., 2004; Broussard et al., 2011).

The cerebellum expresses ERs, ARs, and the synthesizing enzymes for E2 and androgens (Sakamoto et al., 2003; Tsutsui et al., 2011; Hedges et al., 2012). The first evidence of the influence of neural E2 in cerebellar learning has been provided by a study in which synaptic plasticity at the Purkinje cell and the VOR adaptation was examined in ovariectomized mice, in ERβ knock-out female mice, and in male mice, after the administration of E2 (Andreescu et al., 2007). These authors found that E2 had relevant impact on the expression of VOR gain-down adaptation and regulated cerebellar synaptic plasticity influencing PF-LTP. First, it was shown that E2 enhanced the LTP amplitude at the PF–Purkinje cell synapse, while leaving LTD unaffected. Second, in Purkinje cells, ERβ activation by E2 significantly improved the gain-decrease adaptation of the VOR (Andreescu et al., 2007). In a subsequent study, the impact of E2 in the cerebellar synaptic plasticity was examined by pharmacological inhibition of E2 synthesis in male rats both *in vitro* at the PF–Purkinje cell synapses and *in vivo* evaluating the VOR adaptation (Dieni et al., 2018a,b). The application of the P450 aromatase inhibitor letrozole in the flocculus of cerebellar slices prevented the PF-LTP without affecting the PF-LTD impeding the adaptive gain reduction of the VOR. Together with the sex neurosteroid-mediated bidirectional vestibular synaptic plasticity, the cerebellum participates in the visuo-vestibular calibration of the VOR. It is likely that E2 facilitates the gain-increase of VOR by acting at the level of the vestibular system and the gain-down regulation by acting at the level of the cerebellum (Dieni et al., 2018a,b).

## Neural E2 Affects Striatal LTP

Neural E2 exerts an important role in LTP induction in the nucleus striatum with E2 receptors diffusely expressed in the basal ganglia (Creutz and Kritzer, 2004; Krentzel et al., 2019). Electrophysiological experiments performed in neurons of the dorsal striatum of the male rat showed that E2 synthesis and ER activation are required for the induction of LTP in both spiny projection neurons (SPNs) and cholinergic interneurons because the pharmacological inhibition of P450 aromatase or antagonism of ERs completely prevented the LTP induction of SPNs with no effect on LTD or synaptic depotentiation (Tozzi et al., 2015). Because striatal dopamine (DA) release is critical for LTP induction in this brain structure (Calabresi et al., 2007) and based on the evidence that striatal LTP depends on E2 local synthesis, the interaction between E2 and DA in controlling SPNs’ LTP was explored. Tozzi et al. (2015) suggested that the E2 and DA signaling systems converge on the stimulation of the cAMP–PKA intracellular pathway to facilitate LTP induction in striatal neurons via a cooperation between the D1 DA receptor and the ERs (Tozzi et al., 2015). These findings were also supported by experiments showing a possible facilitatory influence of E2 in the dorsal striatum where DA release has been demonstrated to be potentiated by E2 (Song et al., 2019).

## Neural E2 Affects LTP in the Amygdala

17β-estradiol is suggested to play a role in synaptic plasticity also in the amygdala, a brain structure where the presence of P450 aromatase has been reported (Zhao et al., 2007; Bender et al., 2017). The amygdala is considered a core nucleus of “emotional” memory and for the responses to emotion (Pape and Pare, 2010). Accordingly, dysfunction of its neuronal networks is implicated in pathological conditions such as depression and post-traumatic stress disorder (Tovote et al., 2015). Depression-like symptoms have been observed in women under treatment with P450 aromatase inhibitors providing evidence of the importance of the E2 signaling system in the amygdala-related physiology (Gallicchio et al., 2012).

In a recent study, Bender and colleagues (Bender et al., 2017) explored the possible influence of the E2 in the LTP induction of neurons of the basolateral amygdala, a brain region characterized by important synaptic plasticity and by the presence of P450 aromatase both in male and female rodents. Here, the authors found that, beside the effect on the spine density, pharmacological inhibition of P450 aromatase prevented the LTP induction in amygdala slices of female rodents but not in males. The gender-specific role of E2 in LTP of amygdala neurons points to the importance of conducting further studies in the field to better understand the sex-related differences observed in mood disorders and to take into account the side effects of P450 aromatase inhibitors.

## The Role of Circulating Hormones

Whether circulating sex hormones affect the long-term synaptic changes induced by electrical neuronal activity in the brain is an intriguing possibility. E2 synthesized in the CNS may be affected by the estrous cycle in rodent, as reported in the rat hippocampus where neural E2 levels changed according to different phases of the cycle (Kato et al., 2013). Neural E2 changes were suggested to result by indirect access of fluctuating hematic progesterone into the brain and its subsequent conversion into E2 (Kato et al., 2013) or by changes of the phosphorylation status of P450 aromatase depending on fluctuation of different kinases related to synaptic plasticity (Balthazart and Ball, 2006; Hojo and Kawato, 2018). The resulting effect of neural E2 fluctuations appeared to be correlated in the rat hippocampus with the induction of LTP or LTD together with changes of dendritic spine density. In fact, it has been shown that the LTP amplitude changes are depending on the estrum phase in the hippocampus (Warren et al., 1995). Subsequent comparative studies on the occurrence and amplitude of LTP were performed in vestibular neurons of male and female rats (Pettorossi et al., 2011). Specifically, while HFS protocol was able to consistently induce LTP in male rats within seconds (fast-developing LTP), in females HFS protocol produced fast and slow LTP or even LTD. The amplitude and occurrence of LTP depended on the estrous phases, with minor probability to induce fast LTP in proestrus (Pettorossi et al., 2011). This effect might depend on the marked structural changes of the neurons during the estrous cycle, but it might also be a result of the influence of circulating hormones in the synthesis of the neurosteroids. It has been found, in fact, that the acute administration of testosterone that induces LTP in male can induce LTD in female only during the proestrus phase (Grassi et al., 2012). This suggests that in the presence of high levels of circulating E2 (proestrus), the conversion of testosterone into E2 is inhibited, whereas the synthesis of DHT is facilitated.

The interesting data obtained by analyzing the long-term responses in vestibular nuclei prompt to extend this study to the hippocampus by using a similar approach for understanding how memory and learning could be correlated with the expression of the neural synaptic plasticity. Changes in the thresholds of the induction of long-term phenomena might vary with behavioral changes, as reported in EEG experiments in women (Sumner et al., 2018).

## Dendritic Spine Remodeling Is Associated to Hippocampal E2-Dependent LTP

Most of the studies on the role of sex neurosteroids in hippocampal synaptic plasticity demonstrated that E2 facilitates LTP induction and enhances dendritic spine formation. E2 has been reported to increase apical dendritic spine formation in projections that are closed to presynaptic terminals on hippocampal CA1 pyramidal cells. It has been reported that this action is achieved by activation of genomic ERs, most likely the ERα (Gould et al., 1990; Woolley et al., 1990, 1996; Woolley and McEwen, 1993; Murphy and Segal, 1996; Woolley, 1998; Hao et al., 2003; MacLusky et al., 2005). Accordingly, ERs activation is able to either decrease GABAergic inhibition (Murphy et al., 1998; Rudick and Woolley, 2001) and increase NMDAR expression and function (Weiland, 1992; Warren et al., 1995; Gazzaley et al., 1996; Cordoba Montoya and Carrer, 1997; Woolley et al., 1997; Good et al., 1999; Cyr et al., 2000, 2001; Bi et al., 2001; Rudick and Woolley, 2001), ultimately increasing the spine density of neurons. Hippocampal LTP have in fact been correlated to the simultaneous increase of both the spine density and the NMDAR-dependent synaptic transmission (Smith and McMahon, 2005, 2006), suggesting that E2 during LTP formation is able to trigger both morphological and functional changes. Synaptic changes induced by E2 during LTP were reported to affect the structure of neuronal circuitry by increasing the polymerization of filamentous actin proteins in the dendritic spines (Kramar et al., 2009) and by rapidly enhancing their head structure and density via activation of ERα, but not ERβ (Ooishi et al., 2012).

The morphological and electrophysiological changes triggered by E2 during LTP may be part of the same E2-dependent mechanism. Synergic effects consisting of formation of new spines and stabilization of synaptic strength in active mature synapses have in fact been reported to be necessary for long-term synaptic consolidation of neurotransmission (Toni et al., 1999; Yang et al., 2008). However, an alternative possibility considers the functional and structural events happening during E2-dependent LTP formation as two distinct phenomena, according to experiments of acute or chronic inhibition of E2 synthesis (Vierk et al., 2012; Fester and Rune, 2015).

Overall, even if it is possible to hypothesize that initial structural changes of dendritic spines induced by E2 precede a full LTP induction, the idea that neural E2 is able to trigger LTP more rapidly than any possible change of synaptic structure is likely to occur. In this scenario, the induction of LTP of synaptic transmission, involving the increased synthesis or availability of E2 during neuronal activity, might represent the early phase in the process that allows subsequent spine formation and consolidation (Yang et al., 2008; Kato et al., 2013; Dickens et al., 2014).

Interestingly, neural androgens may also increase spine structure suggesting that the machinery at the basis of synaptic plasticity needs structural improvement either for the induction of the E2-mediated LTP and for the induction of androgen-mediated LTD (Leranth et al., 2004; Hatanaka et al., 2009).

Thus, sex neurosteroids are suggested to influence long-term synaptic plasticity by complex mechanisms of action, with estrogens and androgens producing opposite effects on LTP and LTD induction, respectively, and both of them being responsible for the enhancement of dendritic spine formation. This reveals a multifaceted mechanism at the base of neurosteroid influence in synaptic plasticity. In fact, estrogens and androgens have opposite effects in the long-term synaptic events, being the LTP induced by E2 and the LTD by androgens, whereas both neurosteroids enhance dendritic spine formation.

Beside their contribution in synaptic plasticity, the evidence reported in this review of the literature underly the involvement of these molecules in the regulation of several behavioral aspects that are related to the motor system function, to emotional manifestations and cognition.

## Author Contributions

AT and VP conceived and wrote the manuscript. LB reviewed the manuscript. All authors contributed to the figure preparation and critically revised the manuscript.

## Conflict of Interest

The authors declare that the research was conducted in the absence of any commercial or financial relationships that could be construed as a potential conflict of interest.
